# Mixing Performance of a Cost-effective Split-and-Recombine 3D Micromixer Fabricated by Xurographic Method

**DOI:** 10.3390/mi10110786

**Published:** 2019-11-16

**Authors:** Ramezan Ali Taheri, Vahabodin Goodarzi, Abdollah Allahverdi

**Affiliations:** 1 Nanobiotechnology Research Center, Baqiyatallah University of Medical Sciences, Tehran 1435116471, Iran; 2 Applied Biotechnology Research Center, Baqiyatallah University of Medical Sciences, Tehran 1435116471, Iran; v.goodarzi@bmsu.ac.ir; 3 Biophysics Department, Faculty of Biological Sciences, Tarbiat Modares University, Tehran 14115-335, Iran; aallahverdi@gmail.com

**Keywords:** microfluidics, micromixer, Reynolds number, split and recombine, microfabrication, diffusion, lamination

## Abstract

This paper presents experimental and numerical investigations of a novel passive micromixer based on the lamination of fluid layers. Lamination-based mixers benefit from increasing the contact surface between two fluid phases by enhancing molecular diffusion to achieve a faster mixing. Novel three-dimensional split and recombine (SAR) structures are proposed to generate fluid laminations. Numerical simulations were conducted to model the mixer performance. Furthermore, experiments were conducted using dyes to observe fluid laminations and evaluate the proposed mixer’s characteristics. Mixing quality was experimentally obtained by means of image-based mixing index (MI) measurement. The multi-layer device was fabricated utilizing the Xurography method, which is a simple and low-cost method to fabricate 3D microfluidic devices. Mixing indexes of 96% and 90% were obtained at Reynolds numbers of 0.1 and 1, respectively. Moreover, the device had an MI value of 67% at a Reynolds number of 10 (flow rate of 116 µL/min for each inlet). The proposed micromixer, with its novel design and fabrication method, is expected to benefit a wide range of lab-on-a-chip applications, due to its high efficiency, low cost, high throughput and ease of fabrication.

## 1. Introduction

Miniaturization is of great importance in science and technology. Microfluidics focuses on the field of miniaturized fluidic devices fabricated by the methods of microfabrication. Nowadays, numerous applications of microfluidic devices are developed by research groups and technological companies. A wide variety of applications is invented using microfluidic devices, owing to their small length scale, ranging from sub-micrometer to submillimeter scale. Although microfabrication methods have improved to precisely fabricate complex microstructures, they are usually expensive and advanced. Developing cost-effective, but efficient microfluidic devices is substantial to pave the way to mass production of microfluidic devices.

Efficient mixing of fluids is an indispensable task in microfluid platforms, including sample analysis [1], bioreactors [2,3], drug delivery [4] and nanomaterials synthesis [5,6,7]. There are two basic categories of micromixers: Passive and active. Active mixers employ an external power source and use various phenomena to enhance mixing of fluids. For instance, turbulences can be made by means of an external source inside the fluidic channels [8,9,10,11]. Employing active methods requires more complex components integrated into the device. On the other hand, passive micromixers benefit from optimized geometries of the channels. Mixing is a challenging issue, due to the low Reynolds number (Re) of microfluidic systems. The laminar flow regime makes it difficult to generate turbulent flow inside the channels. Consequently, the dominant mass transport mechanism is typically diffusion between the fluid layers instead of chaotic advection. Therefore, designing the channel structures play an important role in maximizing the mixing quality. Moreover, different levels of flow rates may be demanded, depending on the application. Micromixers must be functional over a wide range of Reynolds numbers.

Various methods have been developed to assist passive mixing based on their specific features, briefly listed as: Spiral micromixers [12,13,14], zigzag-shaped channels [15], T-shaped mixers [16], floor-grooved channels [17,18], herringbone mixers [19,20], obstacle-based mixers [21], convergent-divergent walls [22], split and recombine (SAR) mixers [23,24,25,26,27,28,29] and lamination-based mixers [20,30,31]. All of the above-mentioned methods may be used in both planar [12,15,21,22,23,25,28,32] or three-dimensional (3D) [13,14,16,17,18,19,20,24,26,27,29,30,31] systems. 3D passive micromixers typically benefit from spatial structures to generate more effective vortices. Fabrication of 3D devices is generally more complex and more expensive in comparison to planar ones. To study limitations and advantages of above-mentioned mixer types, there are excellent review articles with full details and classifications of passive micromixers provided by Cai et al. [33] and Nguyen et al. [34].

Lamination-based mixers shorten the diffusion path and increase the contact surface between two phases by generating several layers of fluids arranged together. They are classified into two main categories: Parallel lamination [27,30,32], and serial lamination [20,27,31,35] mixers. In the case of parallel lamination mixers, fast mixing is achieved by laminating fluid layers at the inlet of fluids via the specific configuration of the fluid inlets. Thereupon, mixing is enhanced through the laminated stream until the phases completely mix. In the case of serial lamination mixers, the fluid stream (coming from the inlet) is serially split and recombined. The inlet stream divides into two or more sub-streams and they are joined again at the recombining step into one stream as a laminate. The number of sub-streams rapidly increase by repeating this process, which significantly enhances the mixing.

One of the first lamination-based micromixers has been developed by Branebjerg et al. [31]. A plane was repeatedly placed inside the microchannel to split the fluid stream and recombine it with inverse direction to double the fluid layers after each stage. The device was fabricated by anodic bonding of wet etched Pyrex and silicon substrates, which is still an expensive and time-consuming method after more than two decades. Buchegger et al. [32] developed a planar parallel lamination micromixer. Instead of using simple T-shaped inlet, a specific inlet configuration was employed to create a four-layer flow. Mixing time of around 1ms at very low flow rates about 1 µL/min was achieved. Deep reactive ion etching was the main fabrication step to fabricate the device, which demands advanced material and equipment. Sadabadi and coworkers [30] developed a three-layer Polydimethylsiloxane (PDMS) micromixer. The microchip was composed of three PDMS layers fabricated by the conventional soft lithography process. They measured the mixing efficiency by dividing the outlet into four branches and measuring the pH of each branche. Mixing efficiency of 85% was achieved at Reynolds numbers lower than 5.5 (flow rate of 40 µL/min). Lim et al. [35] reported a 3D passive lamination micromixer called crossing manifold micromixer (CMM). Six layers of manifolds were placed horizontally and vertically in a row to rearrange the fluid layers in a 3D manner. The device was highly efficient in comparison to the other lamination-based passive micromixers. Mixing efficiency of 90% was achieved at the flow rate of 3 µL/min within the length of 250 µm. Additive manufacturing was used, due to the geometrical complexity. They utilized sequential photolithography steps and two-photon absorption stereo-lithography processes to fabricate the device. This time-consuming fabrication method requires advanced equipment, which is not a suitable choice for low-cost mass fabrication of the microchips. Kim and coworkers [27] combined two mixing mechanisms, “split and recombine” and “chaotic mixing”, called serpentine laminating micromixer (SLM). They employed F-shaped structures in a two-layer design to laminate fluid layers over each other and make chaotic advections at high-Reynolds flow. High levels of mixing efficiency were achieved by this method. The SLM device was fabricated by polymer inject molding. The mold was fabricated by means of the photolithography of SU8 structures on nickel substrates, followed by electroplating of nickel. This fabrication process is one of the most promising methods of mass production of microfluidic devices. However, it is not a suitable choice for the designing step of the device development, while fabrication flexibility is needed to testify various designs. Tofteberg and colleagues [20] developed a mixer with controlled rotation (90°) of the flow by means of the staggered herringbone structures. The mainstream was serially split into two sub-streams and recombined after the rotation of the sub-streams. This lamination-based mixer was fabricated through micro-milling of polymer substrates and thermal bonding.

Xurography is a rapid fabrication alternative for conventional microfluidic device fabrication methods. This method attracted attention during the last decade for rapid prototyping of microfluidic devices with relatively large feature sizes. The technique employs a razor to transfer the desired design to foils by means of cutter plotter machine, traditionally used in the sign industry. There are informative papers about the limitation, advantages and applications of Xurography technique in the field of microfluidics [36,37,38]. Martinez-Lopez et al. [23] fabricated disposable micromixing arrays based on the Xurography technique. They designed a planar split and recombine mixer with minimum channel width of 500 µm. Fluids were injected by micro-pipetting. Very low Reynolds numbers were tested. Although mixing was tested at very low flowrate, they achieved maximum MI of 65% at Reynolds number equal to 0.07. In the present work, MI of 90% was obtained at Reynolds number of 10, which reflects the high efficiency and throughput of the proposed design over the planar design [23]. Xurography technique is mainly used to fabricate planar geometries. In the case of 3D micromixers, various microfabrication methods have been employed to fabricate 3D mixing structures, such as: Soft lithography [30,39,40], injection molding [27], micro-milling [20,41], additive manufacturing [14,35,42], and etching [31,32]. To the best of the authors’ knowledge, this study is the first work to use Xurography to fabricate and test a 3D micromixer. Integration of 3D structures, fabricated through the method presented in this work, is a suitable choice for mass fabrication of lab-on-a-chip systems.

In the present paper, a novel SAR micromixer was proposed, and its mixing behavior was studied through numerical simulations and experimental tests. The main mechanism employed to enhance mixing of two fluid phases was the lamination of fluidic layers. This was achieved by means of the novel 3D design of mixing units. The rapid and low-cost fabrication method based on the Xurography was utilized to fabricate the device. The proposed fabrication method is a suitable choice for both research and industrial purposes of 3D microfluid devices, due to its simplicity, flexibility and repeatability. Mixing experiments were conducted to quantitatively evaluate the performance of the proposed device, and excellent performance was observed over a wide range of Reynolds numbers (Re = 0.1 to 10).

## 2. Micromixer Design

**Figure 1:**
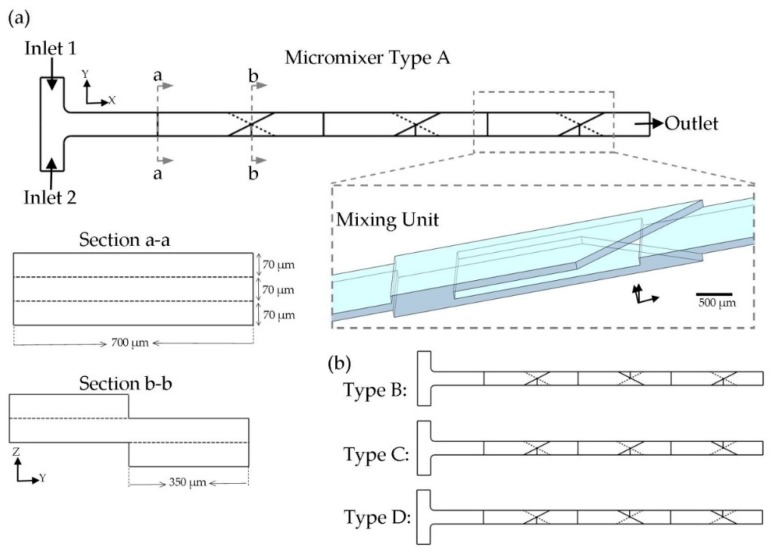
An overview of the geometry of the proposed micromixer. (**a**) Design of the micromixer compromising of three identical mixing units (Type A); 3D view of the mixing unit is illustrated. Furthermore, cross section views of the fluid splitting and recombining zone are illustrated. (**b**) Other three types of mixing units sequence, which was studied in this paper. Dashed lines are used to represent the rear triangle.

The main idea of the present work is to achieve the maximum mixing performance in a limited area by taking advantages of the 3D design. As discussed in the introduction, many geometrical designs have been developed for mixing purposes. In this study, splitting the fluid in the z-direction and recombining the fluid stream in a 3D manner was used as the micromixer configuration. The proposed design for each SAR mixing unit is shown in Figure 1a. Each mixing unit consisted of triangular-shaped crossing channels, which were located in the opposite direction on the top and bottom of the main channel. This junction was used to recombine the fluid sub-streams, which were split in the upstream into two parallel channels located over each other. As illustrated in the cross section views in Figure 1a, the fluid stream experienced no lateral movement during the splitting process in the Z-direction, but the fluid arrangement was disordered by joining fluid layers together in the XY-plane (during the recombining step). By applying the present design, the mixing performance was greatly augmented in comparison to a straight microchannel with the same cross section (700 × 70 µm^2^). It is worthwhile to mention that dimensions of the channels were determined to the fabrication limitations. Xurography was used as the fabrication method. Thereupon, the thickness of the fabricated channel was fixed, equal to the adhesive layer’s thickness (70 µm). Based on our experiments, there is a minimum size (700 µm) for the width of the channel by employing the present fabrication method and equipment. As a result, it was not feasible to achieve cross section Aspect Ratios (AR) higher than 1 to 10. It has been proved that, by increasing the AR, there would be a larger area for the mass transfer [43,44]. Due to the laminar flow condition, which prevails here, fluid streamlines were parallel in a straight channel, and a negligible mass transfer could happen in the fluids’ interface. To compensate this issue, the present design reshaped the fluid streamlines inside the channel, which yielded to more mass transfer rate dominated by diffusion. In this way, the fluid exchange happened through the laminated fluid layers coming from the top and bottom. This novel junction configuration was used to improve the molecular diffusion by decreasing the diffusion path and increasing the contact surface between the two phases. In addition, the geometry was designed to decrease the chance of air bubble trapping (during the device filling process) by preventing sharp and concave corners.

A T-shaped inlet design (with the width of 700 µm) was used to inject two fluid species into the mixer. The whole mixer geometry consisted of three mixing units. This number was estimated to be suitable, based on our experiences. It is worthwhile to consider that mixing units may be repeated with different orientations. Two orientations of the triangles are possible for a mixing unit, which are symmetrical shapes about the ZX-plane. For a micromixer compromising of only one mixing unit, the same mixing efficiency would be achieved by passing the fluids over the unit with the same test conditions. However, for a row of mixing units connected together, the concentration distribution of the fluid leaving a unit affects the next unit’s mixing performance. Thereupon, the sequence of mixing units is crucial to the micromixer design. The total number of eight combinations are available (two orientations for each mixing unit and three mixing units). We studied four patterns, because other probable combinations were symmetrical geometries about the XY-plane, and no difference in the mixing quality of the output may be observed, Figure 1b.

## 3. Materials and Methods

### 3.1. Numerical Analysis

Numerical studies on the fluid flow and mixing were carried out by means of a lab-made code based on the finite element method. Numerical simulations indicated a high mixing efficiency of the proposed scheme. In order to model the function of the micromixer device, the flow field was first solved for the whole domain. Next, the concentration distribution inside the micromixer was obtained, due to the flow and boundary conditions. Finally, MI after each mixing unit was investigated, to be compared to the experimental results.

Due to the physics of the problem, Navier-Stokes and continuity equations were solved for a laminar, incompressible and Newtonian flow in the steady-state mode:

(1) $$\rho \left[\left(\vec{V}\cdot \nabla \right)\vec{V}\right]=-\nabla p+\mu \nabla^{2}\vec{V},$$

(2) $$\nabla \cdot \vec{V}=0,$$

where $\rho \;\left(\mathrm{kg}/m^{3}\right)$, $\mu \;\left(N\cdot s/m^{2}\right)$, $\vec{V}\;\left(m/s\right)$ and p (Pa) are the fluid density, fluid dynamic viscosity, vector of flow velocity and pressure, respectively. A pressure-driven flow was solved, and no-slip boundary conditions were set for the walls. The boundary condition of fully developed laminar flow with a parabolic velocity profile was selected for two inlets. Atmospheric pressure was set at the outlet. As two solutions of dye in water were used as working fluids during the experiments, the hydrodynamic properties were considered to be similar to those of water. Therefore, density and dynamic viscosity of the water-dye mixture was assumed to be $1000\;\mathrm{kg}/m^{3}$ and $10^{-3}N\cdot s/m^{2}$, respectively.

After obtaining fluid velocity and pressure, the convection-diffusion equation was used to model the mass transport [44]:

(3) $$D\nabla^{2}C=\left(\vec{V}\cdot \nabla \right)C,$$

where $D\;\left(m^{2}/s\right)$ and $C\;\left(\mathrm{mol}/m^{3}\right)$ denote the diffusion constant and concentration, respectively. Pure water (mass fraction = 0) and water-dye mixtures with the concentration of $1\;\mathrm{mol}/m^{3}$ (mass fraction = 1) were introduced to the inlets. The diffusion constant was assumed to be in the order of the species diffusion constant in the water at 25 °C. Therefore, diffusion constant was set to $10^{-9}{\;m}^{2}/s$, which has been widely used by other research groups in the case of solutions in water [13,34].

Equations were solved using the generalized minimal residual method (GMRES) iterative solver. The convergence criterion employed in this numerical solution was the root-mean-square residual value of less than 10^−6^. Tetrahedral mesh was used for the entire domain, due to the complexity of the geometry. Mesh refinements were applied to capture the flow boundary layer and concentration gradients. Maximum element size was set to 5 µm through the whole geometry. Therefore, a number of mesh elements was always more than ~1,700,000 for each mixing unit. Mesh size was optimized based on the mesh independency test on the distribution of velocity magnitude and concentration. P1+P1 and cubic discretization were used for fluidic and mass transport elements, respectively. Based on our experience, this method showed robust results. The proposed scheme was compared to a simple T-type micromixer with a similar channel length, height and width (divided into ~ 4,500,000 3D structured mesh elements).

To investigate the mixing performance of the mixer, a quantitative method was needed. The mixing index (MI) was calculated at the region of interest, defined as bellow [34]:

(4) $$\mathrm{MI}=1-\sqrt{\frac{1}{N}\overset{N}{\underset{i=1}{\sum }}{\left(\sigma_{i}\right)}^{2}},$$

where, N is the number of sampling points in the region of interest and $\sigma_{i}$ is the total deviation at the sampling point, defined as bellow:

(5) $$\sigma_{i}=\frac{c_{i}-c_{\mathrm{mix}}}{c_{\mathrm{unmix}}-c_{\mathrm{mix}}},$$

where $c_{i}$, $c_{\mathrm{mix}}$ and $c_{\mathrm{unmix}}$ denote concentration at the sampling point, expected concentration with perfect mixing and no mixing, respectively. MI varies from 0 to 1, while the value of 0 represents no mixing, and 1 indicates complete mixing.

Dimensionless parameters are widely used to indicate the working condition of a micromixer. Reynolds number reflects the ratio between the momentum and viscous forces, defined as bellow:

(6) Re=ρUDhμ,

where, ρ, μ, $D_{h}$ and U is the fluid density, fluid dynamic viscosity, hydraulic diameter of the channel and average velocity of the fluid at the microchannel, respectively. The parameters were calculated at the main channel, which has a rectangular cross section. The hydraulic diameter is defined as bellow:

(7) $$D_{h}=\frac{2WH}{W+H},$$

where, W and H are the width and height of the microchannel. In the present study, the Reynolds number was adequately low that the fluid flow was always in the laminar flow regime.

### 3.2. Fabrication Method

**Figure 2:**
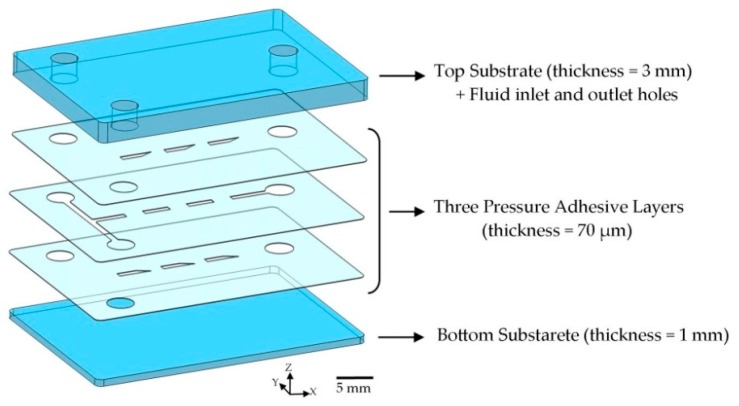
Explosive view of the fabrication method. Three patterned pressure sensitive adhesive (PSA) layers are sandwiched between two cut PMMA substrates shaping the microfluidic device (type A). Three PSA layers built the 3D micromixer geometry, as illustrated in Figure 1. Inlet and outlet holes were suited on the upper substrate and three adhesive layers to allow fluids to enter the micromixer.

The microfluidic device was made by sandwiching the composing layers, Figure 2. Three pressure sensitive adhesive (PSA) layers were sandwiched between two Polymethyl Methacrylate (PMMA) substrates to form the mixer. PSA layers have been used to fabricate microfluidic devices with the biomedical application [45,46,47]. The materials were chosen because they were transparent, cheap and easy to fabricate. Two standard commercial PMMA sheets with a thickness of 3 and 1 mm were cut by the conventional laser cutting procedure to form the upper and lower substrates. The inlet and outlet holes with a diameter of 3 mm were suited on the top substrate in their predicted position. According to the design, microchannel patterns were obtained by cutting the fluidic layers by means of a cutter plotter machine (GS24, Roland, Osaka, Japan) on a PSA sheets (FLEXcon, Spencer, MA, USA) with a thickness of 70 µm. The plotting step was divided into two steps, and the forces of 30 and 35 gram-force were exerted on the razor, respectively. This showed more dimensional precision in comparison to the one-step method, based on our experience. Then, chip components were aligned together using a manual lab-made aligner in the x and y directions, plus the rotation (theta). Two steps of alignment were required, due to the 3D design of the device. Despite the fact that Xurography method is fast and cost-effective, dimensional errors are possible during the fabrication process, especially during the alignment process. Maximum dimensional error of ± 50 µm was detected. Based on our experience, this dimensional error was too small to affect the device performance. To completely eliminate the chance of dimensional error during the assembly step, it is possible to use motorized aligner or fabricate solid guides. Afterwards, the device was sandwiched using a lab-made adjustable clamp (shown in Figure S1) for 10 min, and a force of 0.2 kN was made to prevent any leakage. Two cylindrical Polydimethylsiloxane (PDMS) parts were clamped over the inlet holes to connect the fluidic tubes. All the fabrication steps could be completed by a single person in less than 20 min.

### 3.3. Experiment Procedure

For experimental analysis, the device was put under the microscope (Motic B2-320, Motic Co., Chengdu, China) to monitor the mixing behavior. A digital camera (Nikon D5600, Nikon Co., Tokyo, Japan) was mounted on the microscope to capture microscopic images of the channels. Experiments were carried out by injecting fluid samples through tubing using an adjustable syringe pump (SP110 HPM, Fanavaran Nano-meghias, Tehran, Iran). A flow rate range of 1.16–1160 μlit/min was set for each inlet, resulting in a Reynolds number range of 0.1–100 in the main channel. Yellow and blue 20 %wt solutions of commercial food dyes were prepared by diluting in deionized water, used as working fluids. Tests were repeated for three times, and the average values of MI are reported.

### 3.4. Evaluation of Mixing Performance via Optical Methods

Precise and repeatable evaluation of the experimental MI is of great importance to show the device ability and validate numerical simulations. To calculate the experimental MI, an optical method was employed to measure the standard deviation of the concentration in the region of interest. Various types of fluids have been employed to conduct experimental tests on micromixers, which need a quantitative method to be analyzed. Some groups used microparticles [48] or measuring species concentration or pH of outlets [30,49] to evaluate the mixing quality, which is not the case of the present study. Mixing two fluorescent and non-fluorescent fluids have been employed as another practical method [20,21]. Since the light intensity is proportional to the mass fraction of the fluorescent fluid, MI can be evaluated just by optically measuring the light intensity. This method has its limitation instead of its simplicity. Using fluorescent fluids is expensive, which is in contrast with the concept of the present study. Besides the mentioned working fluids, using colored water is a convenient choice. To do so, a mixture of dyes and pure water is mainly used by research groups [15,23,41]. Some use pure water and colored water and some used two different colored fluids to be mixed in the micromixer device.

To evaluate the mixing performance, microscopic images were transferred to the image processing software (ImageJ, NIH, Bethesda, MD, United States) and converted to the 8-bit format. Afterwards, primary color (red, green and blue) intensities were extracted over the pixels of the region of interest, and the MI was calculated according to Equation (4). Identical optical test conditions were applied for the experiments to obviate optical measurement errors. Progress of mixing efficiency after each mixing unit was observed in the main channel at a distance of 0.5 mm after the mixing unit.

The conventional method to investigate the mixing efficiency is the grayscale method, based on converting the RGB (Red-Green-Blue) images to grayscale images and performing the pixel color intensities calculations on it. The other method, introduced by Tsai and Lin [15], uses only on the G-component to find a correlation between the reference experimental MI and the G-component. This method is applicable in the case of using blue and yellow dyes as working fluids.

The grayscale method is widely used for experimental calculation of MI, especially where two different colorants mix together and form a new color [50]. This method is based on the transformation and normalization of an image from the RGB system to the grayscale-corresponding image. This action can be easily performed via using the available digital soft wares. The RGB color model is a system in which color of any pixel is divided into three components of Red, Green and Blue. RGB components are a number between 0 and 255 (8-bit data). Full description of this method is provided by Lee et al. [39].

**Figure 3:**
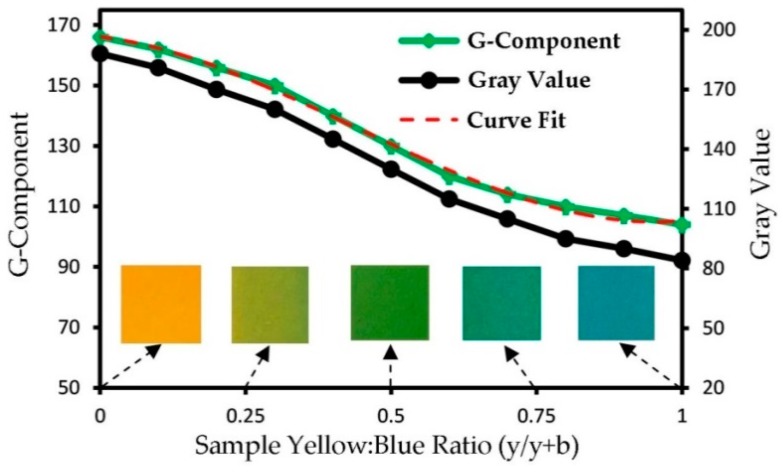
Calibration of the green component and equivalent gray value for different sample concentration ratios (yellow to blue dye). Images of five ratios (from pure yellow to pure blue) are illustrated on the chart. A curve was fitted to find a practical correlation, used during the image-based MI assessment.

As the yellow and blue colorants were utilized, which turn into green, while mixed, the green intensity may be selected to be studied among the RGB pixel intensities. Experimental tests were needed to find a calibration equation between the G-component and the real mass fraction of the fluid at the region of interest. To do so, 11 standard samples of different concentrations of blue and yellow fluids were prepared in macroscale. Then, they were introduced to the microfluidic device, and related images were captured. For each sample, the mean RGB components were obtained using ImageJ software, shown in Figure 3. Furthermore, images were transferred to grayscale, and the grayscale values were obtained too.

Both methods showed an acceptable correlation. The G-component method was selected to analyze the experimental tests, because this method was validated by Tsai and coworkers [15] in the case of using blue and yellow solutions as working fluids. A third degree polynomial curve was fitted on the G-component curve as:

(8) $$G=114.6x^{3}-151.6x^{2}-23.8x+166.1,$$

where, G and x denote the G-component (0 to 255) and a real mass fraction (yellow:blue mass ratio, 0 to 1) at the region of interest, respectively. To estimate the MI, a one-to-one mapping of data gathered from the image processing and the species concentration was needed. Therefore, the inverse function of the Equation (8) was solved as:

(9) $$x=-8\times 10^{-6}G^{3}+0.0034G^{2}-0.47G+22.7,$$

while x is a number between 0 and 1 (0 for pure yellow dye and 1 for pure blue dye). This equation is not valid for other experiments with different devices, test conditions and working fluids.

## 4. Results and Discussion

This section presents the results of experimental and numerical studies conducted to evaluate the proposed mixer’s performance. First, the hydraulic behavior of the proposed system was investigated based on the numerical simulations and experimental tests. The basic principle of the present geometry was to split and recombine two fluid streams, coming from inlets, in the way that the streamlines reshape to increase the mass transport rate. This was done by using the specific geometry of the splitting zone and recombining zone of the mixing unit, shown in Figure 1. Then, the results of simulation of the mixing performance are presented. As experimental tests were done under an optical microscope, only 2D views of the micromixer were accessible. Numerical simulations were needed to investigate the 3D concentration distribution inside the channels. Above-mentioned results are presented for the micromixer geometry type A (illustrated in Figure 1). Afterwards, the impact of the fluid flow rate (Re) and geometry type were studied after each mixing unit, and relevant experimental and numerical results are compared here.

### 4.1. Experimental and Numerical Results of Micromixer Performance

**Figure 4:**
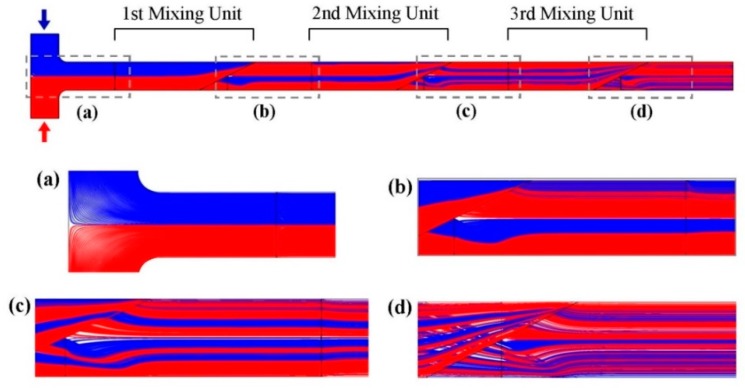
Generation of sub-streams represented by flow streamlines starting from inlets (blue and red). Streamlines are calculated with regard to velocity vectors obtained from the fluid flow simulation. Total number of 500 streamlines with a uniform density of starting position is plotted for each inlet. Due to the novel geometry of the mixing units, streamlines mix together as magnified views are illustrated for: (**a**) T-shaped inlet, (**b**) after passing through first mixing unit, (**c**) second mixing unit and (**d**) third mixing unit.

Physical combination of two fluid streams is crucial to enhance the mixing. The specific design of the 3D mixing units allowed the fluid layers to physically mix together. The proposed design is examined in this section. Due to the laminar flow regime, two fluid streams had no significant diffusive mass transport at the T-shaped inlet. After the inlet, the main channel was divided into two parallel channels and recombined together again. During the recombination, two top and bottom streams were placed together in the lateral direction. Streamlines were simulated to observe this phenomenon. Starting positions of the streamlines had a uniform density at two inlets, and they were simulated for the entire geometry, illustrated in Figure 4. The number of fluid layers (shown in blue and red) was doubled after each mixing unit (Figure 4a–d). Consequencently, the number of fluid sub-streams increased to 4, 8 and 16 after the first, second and third mixing unit, respectively. It is worthwhile to note that the streamlines had a 3D distribution, but only the 2D view is shown in Figure 4, thus, they do not seem symmetric. Altering the flowrate did not change the streamlines pattern, due to the laminar flow. Note that this is different from the mass transport. In fact, behavior of the fluid flow affects the mass transport behavior, but not vice versa.

**Figure 5:**
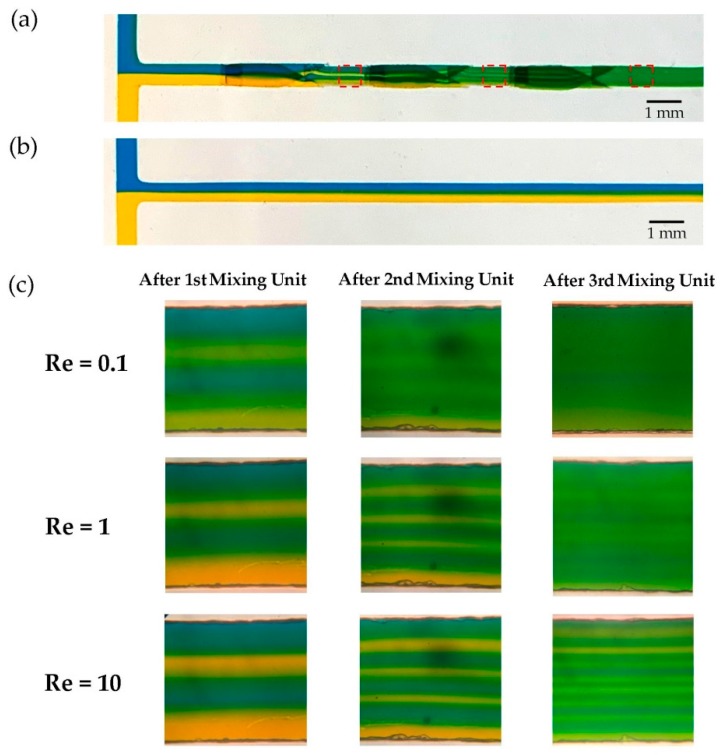
Experimental results of the micromixer performance. (**a**) Macroscopic image of the proposed device, mixing blue and yellow dyes at Re = 1. The fluid layers mimicked the pattern predicted by the numerical simulations, illustrated in Figure 4. Fluid layers were generated as expected. A slight misalignment was caused during the fabrication step. Based on our experience, the device had robust output against it and performed well. (**b**) A straight channel was fabricated with the same channel width, height and length. It was tested with the same test condition as (**a**). Two phases were lightly mixed at the outlet in the absence of 3D mixing elements. (**c**) Microscopic images of the main channel after three mixing units for three different Reynolds numbers (Re = 0 1, 1 and 10) show that an incomplete mixing was captured at the outlet for high flow rate (Re = 10). In contrast, more efficient mixing was achieved in the case of lower fluid flow rates. Images show the region at the distance of 0.5 mm downstream of mixing units, where MI is calculated.

Results of the streamline simulation were validated by means of experimental tests. Tests were conducted regarding the procedure described in the experiment procedure section. Microscopic images of the main channel are shown in Figure 5 for three different Reynolds numbers (Re = 0.1, 1 and 10) at the distance of 0.5 mm downstream of three mixing units (red rectangles), where MI was measured. Obviously, the proposed design had an excellent mixing performance in comparison to the straight T-type micromixer with the same test conditions, Figure 5a,b. The pattern of streamlines was similar to what predicted by the numerical simulations, i.e., initial fluid stream (two blue and yellow layers) divide into doubled sub-streams after passing each mixing unit. For higher Re, blue and yellow layers had more distinct borders, and fluids were not completely mixed at the output, Figure 5c. Because two fluid species had less residence time for diffusion of species, due to high velocity. In contradiction, approximately complete mixing was observed at the outlet at Re = 0.1.

### 4.2. 3D Evaluation of Mixing Process

**Figure 6:**
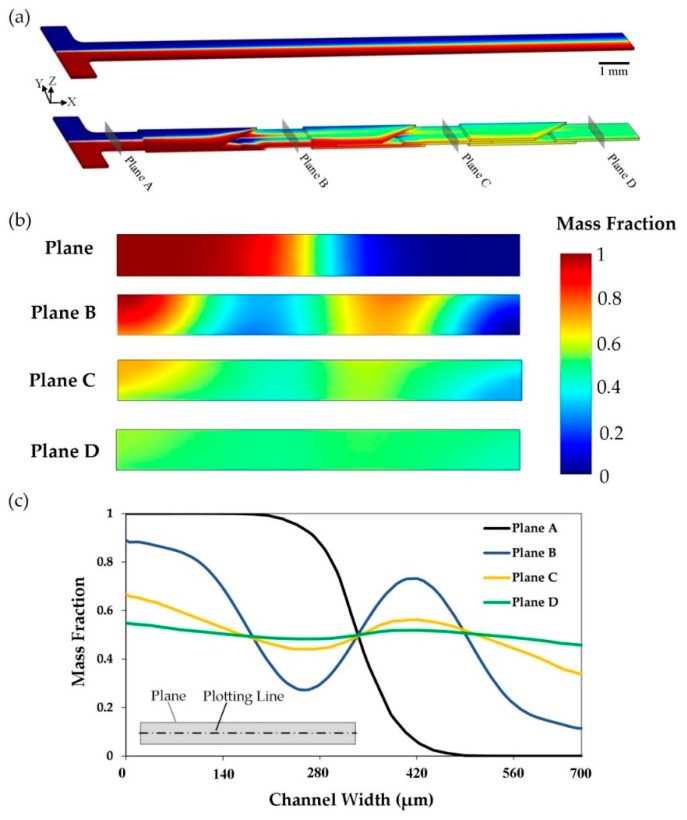
Numerical simulation of the mixing process (micromixer type A). (**a**) Comparison of mixing performance of the proposed design and a straight channel with same cross section size and boundary conditions (Re = 0.1). (**b**) Mas fraction distributions at the inlet and after each mixing unit are illustrated. Location of the planes is shown on the micromixer. (**c**) Plot of mass fraction along an imaginary line middle of the above-mentioned planes. Schematic position of the plotting line is illustrated.

Simulation of the mixing process was performed as described earlier. The sole way to analyze the 3D distribution of concentration inside the geometry was numerical modeling. Diffusion-convection equation was solved for whole geometry to understand the mixing performance in a 3D manner (results are shown in Figure 6). Mass fraction (a number between 0 and 1) was chosen to plot the concentration distribution, due to its simplicity to interpret and compare the charts. A straight channel was modeled and compared to the proposed design of the micromixer (type A) with the same boundary condition and flow rate (Re = 0.1), Figure 6a. The proposed design had an excellent performance in mixing two phases in comparison to the straight one. In the case of the straight channel, the only diffusion in lateral direction was the cause of mass transport. The proposed mixer had the same performance before reaching the first mixing unit. However, the novel design of the SAR mixing units caused the fluid layers to quickly mix together after passing and enhanced the diffusive mass transport. Therefore, the outputs were totally different. Moreover, the mass fraction was plotted in four planes perpendicular to the main channel direction, shown in Figure 6b. Fluid phases were approximately unmixed at plane A. Deviation of the mass fraction along the planes’ area decreased by passing through mixing units until an approximately uniform mass fraction distribution was achieved at the outlet (Plane D). MI was calculated to be 0.09, 0.48, 0.83 and 0.96 at Plane A, B, C and D, respectively. In addition, mass fraction distribution inside the plane on an imaginary line located in the middle of the planes is plotted in Figure 6c. The curves were excellently smooth, which reflects that an appropriate mesh study was performed. Due to the symmetric geometry of the micromixer, curves were symmetrical about the center point of the plane. As expected, mass fraction values converged to 0.5 (Mean of the inlet mass fractions, 0 and 1) by the progress of the mass transport along the micromixer.

### 4.3. Impact of Reynolds Number on Mixing Efficiency

**Figure 7:**
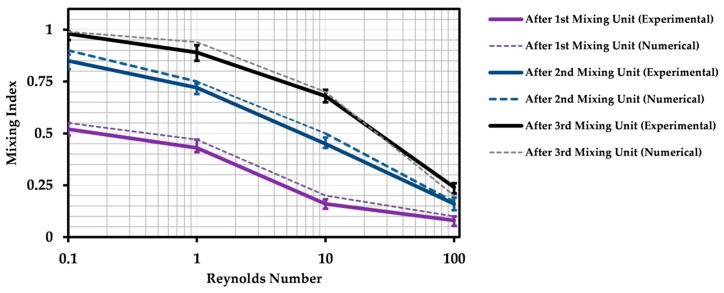
Variation of the experimental and numerical MI after each mixing unit versus Reynolds number (Re = 0.1, 1, 10 and 100) in logarithmic scale. Maximum MI was achieved at lower Reynolds numbers, due to more mass transport caused by molecular diffusion.

Evaluation of the micromixer efficiency over a range of Reynolds numbers is of great importance to find the functional condition of the device. Furthermore, it is an important criterion to compare the present design to other studies. For this reason, results are presented by means of the Reynolds number, which is a dimensionless number. Instead of mixing length or time, experimental results of the mixing quality are presented by means of the MI, calculated after each mixing unit regarding the method described earlier. The region of interest to measure the MI was a rectangle with a width of 700 µm (equal to channel width) and a length of 700 µm. Location of the region of interest was 0.5 mm downstream of the mixing units. The length between the mixing units was chosen with regards to the technical issue of capturing microscopic photos. It is usual to capture photos with a distance from the channel structures to avoid any optical errors. Therefore, the total length of the micromixer was the minimum accessible value, which was minimized to achieve the shortest mixing time. Tests were conducted with respect to the procedure explained earlier in the section of the experiment procedure. Four Reynolds numbers from a different order of magnitudes (Re = 0.1, 1, 10 and 100) were set for tests. This range covers most of the similar researches published by other groups on the SAR micromixers [25,28,30,39,51,52]. Figure 7 shows the variation of the MI after three mixing units for Reynolds numbers from 0.1 to 100. Mass transport is dominated by molecular diffusion and residence time at Re = 0.1. Channels length was enough to achieve a nearly complete mixing at the outlet after the third mixing unit (MI > 0.95). Tests were repeated for three times, and average values and corresponding error bars are plotted. Maximum error of 7.5 % was observed within all the tests, which was mainly related to the image processing step independent of the flow rate. By increasing Re, values of MI continuously decreased, which reflects the fact that mass transport is reduced, due to shorter residence times at high Reynolds numbers. MI rose after each mixing unit, in a fixed Reynolds number. In addition, MI growth was more after passing the second mixing unit compared to the third one. Similarly, this was observed during the numerical simulation, Figure 6. Because the potential of diffusion-based mass transport decreased by passing through each mixing unit. Mass flux is proportional to the difference of concentration, according to Fick’s law. Therefore, the concentration gradient (difference of maximum and minimum of the mass fraction on the planes in Figure 6b) decreased after each mixing unit yielding to less mass flux and MI growth. The simulated data are plotted too. Good agreement between the experimental and numerical results was observed. Among all of the results, the maximum difference between the experimental and numerical result was 9%.

It is worthwhile to mention that the main mechanism of the present design is to form thin layers of two fluids laminated together (shown in Figure 4 and Figure 5). Thereupon, two fluids interface, needed for diffusion, increases significantly. Many SAR micromixers developed by research groups use the splitting and recombining process to enhance the chaotic advection by the generation of secondary flows using the geometrical features at intermediate and high Reynolds numbers [15,21,25]. In those cases, MI is near to 1 at very low flow rates and drops down by increasing the Re, which causes less molecular mass transport at low Reynolds number range. However, The MI values increase again after the above-mentioned drop, due to rise of the momentum effects. Here, the geometry is designed with no obstacles or sharp deformations. Lamination was caused by fluid flow. In consequence, curves plotted in Figure 7 are continually descending, in contradiction to the afore-mentioned behavior and similar to what reported about the micromixers benefiting from lamination method [30]. Note that, the chart is plotted in logarithmic scale. If plotted in linear scale, the shape of the curves would be concave up and descending.

### 4.4. Impact of Geometry Type on Mixing Efficiency

**Figure 8:**
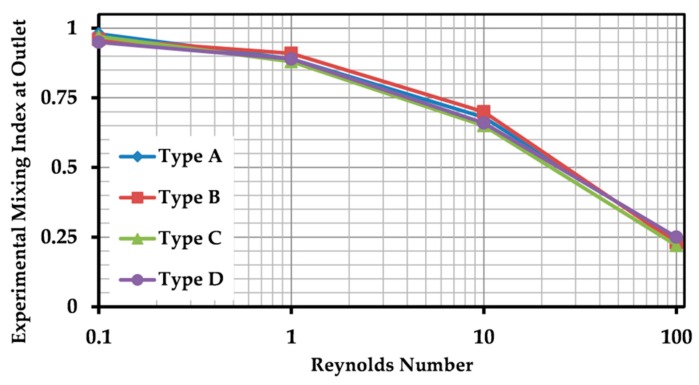
Impact of geometry type of the micromixer (orientation of the sequence of three mixing units) on the mixing performance through MI measurements. Experimental data are presented for a range of Reynolds numbers and four micromixer designs, illustrated in Figure 1.

Four geometry types, presented in Figure 1, were examined to study the impact of the combination of the mixing units with different orientations. No significant difference was observed. To do so, fabricated micromixers were tested at four different Reynolds numbers (Re = 0.1, 1, 10 and 100), and MI was calculated at the outlet regarding the method described earlier. Experimental results are shown in Figure 8. The orientation of the mixing units did not affect the pattern of fluid laminations pattern after each mixing unit, because two orientations were symmetrical and divided the main stream into two equal sub-streams. Therefore, for all types of geometry, the layers were generated similarly, and approximately equal MI was observed, regardless of the Reynolds number. No considerable difference was detected at lower Reynolds numbers (Re = 0.1 and 1). There was variation percentage of 8% and 21% among the MI values at Reynolds numbers of 10 and 100, respectively, which were mainly originated from the experimental errors.

## 5. Conclusions

In the present paper, a novel lamination-based split and recombine micromixer is investigated through experimental tests and numerical simulations. Rapid and low-cost method of Xurography was employed to fabricate the multi-layered device. Numerical simulations were performed to analyze the hydraulic and mass transport behavior of the mixer, which were validated by experimental results. Experiments conducted for a wide range of Reynolds numbers, from 0.1 to 100. Four different arrangements of mixer designs were put under test and compared, which had nearly similar mixing performance. According to the results, the main mass transport mechanism of the proposed mixer was diffusion between the layers of the two fluid phases, instead of advection. Generation of the fluid layers was predicted numerically and observed experimentally. Mixing characteristics of the mixer were evaluated quantitatively using image-based MI measurements. The device had mixing efficiencies of 96% and 90% at Reynolds numbers of 0.1 and 1, respectively. MI value of 67% was measured at a flow rate of 116 µL/min (Re = 10), which is a considerable throughput. The advantage of the presented mixer is ease of fabrication, high throughput and functionality over a wide range of Reynolds numbers. The results established the proposed mixer as a feasible candidate to be used for mass fabrication of 3D microfluidic mixers for research and industrial purposes.

## Supplementary Materials

The following is available online at https://www.mdpi.com/2072-666X/10/11/786/s1, Figure S1: Image of the lab-made clamp used to adjust the exerted force on the microchips during the fabrication process.
