# Supplementary material for: Mixing Performance of a Cost-effective Split-and-Recombine 3D Micromixer Fabricated by Xurographic Method
Source: Micromachines (Basel). 2019 Nov 16;10(11):786. doi: 10.3390/mi10110786 (PMC6915444; doi:10.3390/mi10110786)
Supplement: Supplementary file 1 [file micromachines-10-00786-s001.pdf]

micromachines

Article

Mixing Performance of a Cost-effective Split-and-Recombine 3D Micromixer Fabricated by Xurographic Method

Ramezan Ali Taheri [1,*], Vahabodin Goodarzi [2] and Abdollah Allahverdi [3]

[1] Nanobiotechnology Research Center, Baqiyatallah University of Medical Sciences, Tehran 1435116471, Iran
[2] Applied Biotechnology Research Center, Baqiyatallah University of Medical Sciences, Tehran 1435116471, Iran; v.goodarzi@bmsu.ac.ir
[3] Biophysics Department, Faculty of Biological Sciences, Tarbiat Modares University, Tehran 14115-335, Iran; aallahverdi@gmail.com
* Correspondence: taheri@bmsu.ac.ir; Tel.: +989126110865

Received: 1 October 2019; Accepted: 14 November 2019; Published: date

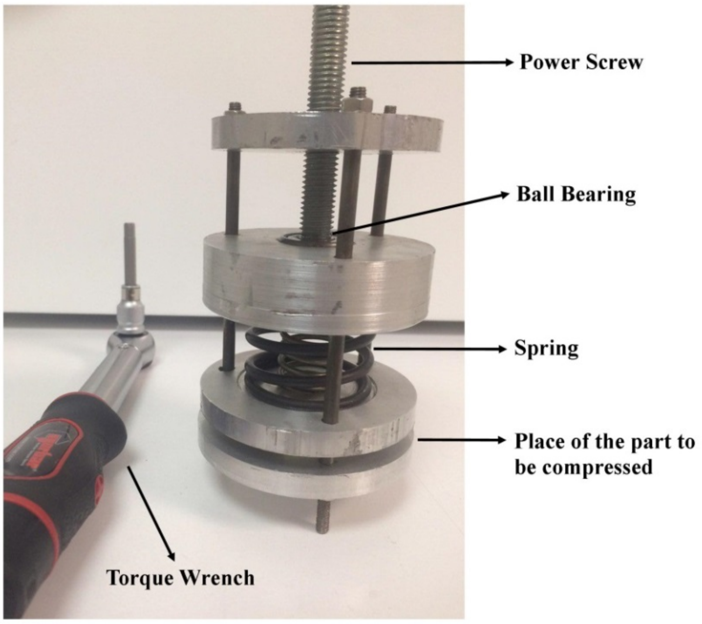

Figure S1. Image of the lab-made clamp used to adjust the exerted force on the microchips during the fabrication step. The idea is to use spring compression to linearly control the exerted force. A power screw is utilizes to convert the rotary motion to linear motion. As the spring had a fixed spring constant, the applied force was adjustable. A torque wrench was used to precisely adjust the screw force. The calibration curve of the clamp force and the applied torque was previously obtained using a digital force sensor.
